# Age-enhanced MAGIC algorithm predicts mortality in pediatric aGVHD: a multicenter study

**DOI:** 10.3389/fimmu.2025.1660861

**Published:** 2025-09-12

**Authors:** Na Song, Hao Xiong, Ri Xu, Wen-Geng Cheng, Shan He, Shaoyang Deng, Benshan Zhang, Dao Wang

**Affiliations:** ^1^ Department of Hematology/Oncology, The Affiliated Children’s Hospital of Xiangya School of Medicine, Central South University (Hunan Children’s Hospital), Changsha, China; ^2^ Department of Hematology, Wuhan Children’s Hospital, Tongji Medical College, Huazhong University of Science and Technology, Wuhan, China; ^3^ Beijing BFR Gene Diagnostics, Beijing, China; ^4^ Department of Pediatric Hematology and Oncology, The First Affiliated Hospital of Zhengzhou University, Zhengzhou, China

**Keywords:** acute graft-versus-host disease, allogeneic hematopoietic stem cell transplantation, non-relapse mortality, MAGIC algorithm, pediatric, risk stratification

## Abstract

**Introduction:**

Acute graft-versus-host disease (aGVHD) is a major contributor to non-relapse mortality (NRM) in pediatric patients undergoing allogeneic hematopoietic stem cell transplantation (allo-HSCT). Although the MAGIC algorithm has been validated in adults, its predictive value in children remains insufficiently explored.

**Methods:**

We conducted a prospective multicenter cohort study including 105 Chinese pediatric allo-HSCT recipients diagnosed with aGVHD between May 2019 and August 2023. Endpoints were 6-month NRM, overall survival (OS), and Day-28 treatment response. Multivariable analyses incorporated clinical variables together with the Panel 2 score, hereafter referred to as Panel 2,using Cox regression for NRM/OS and logistic regression for treatment response.

**Results:**

Age ≥12 years (hazard ratio 4.36, 95% CI 1.62–11.75; P=0.003) and a high Panel 2 score (HR 3.09, 95% CI 1.08–8.82; P=0.035) were independent predictors of 6-month NRM and OS. The high-risk (HR) group, defined by the combination of age ≥12 years and a high Panel 2 score, had markedly higher NRM than the low-risk (LR) group (71% vs 12.2%; HR 5.00, 95% CI 1.75–9.56; P=0.001) and significantly worse OS (P<0.001). Panel 2 was also predictive of Day-28 treatment response, with lower CR/PR rates in the high versus low group (62% vs 92%; P<0.001).

**Discussion:**

The Panel 2 score effectively predicted NRM, OS, and treatment response in pediatric aGVHD. Incorporating age ≥12 years further enhanced risk stratification, enabling clear separation between HR and LR groups. These findings support the potential clinical utility of this combined model and warrant validation in larger, international pediatric cohorts.

## Introduction

Allogeneic hematopoietic stem cell transplantation (allo-HSCT) is a potential curative approach for numerous pediatric patients with both malignant and nonmalignant conditions. Despite advances in transplant techniques and supportive care, acute graft-versus-host disease (aGVHD) remains a severe complication and a substantial cause of morbidity and non-relapse mortality (NRM). Moderate-to-severe aGVHD affects approximately 13 - 47% of pediatric allo-HSCT recipients and is associated with poor outcomes (1). Conventional prognostic approaches—based primarily on clinical grading at aGVHD onset together with standard transplant-related parameters—have limited accuracy in predicting critical outcomes such as NRM and overall survival (OS). This limitation can result in overtreatment of low-risk patients and undertreatment of high-risk patients. Consequently, there is growing interest in developing objective, biomarker-based tools that enable earlier and more accurate risk stratification (2, 3).

For more than a decade, high-throughput detection methods (e.g., proteomic mass spectrometry, cytomics assays, multiplex immunoassays, and array-based assays) have uncovered numerous biomarkers related to aGVHD, significantly advancing the understanding of the complex pathophysiology of aGVHD (4, 5). These biomarkers have been investigated as individual markers (6–8), in composite panels (9), and in biomarker algorithms (10–12). Recent studies have also explored integrating biomarker profiles with clinical and transplantation-related factors using machine learning, offering an expanded approach and representing one of the emerging trends in risk prediction for allo-HSCT recipients (13).

Years of research have progressed from initially screening numerous candidate biomarkers to recently focusing on a few key proteins. Several core markers have since emerged as particularly relevant in acute GVHD. These include soluble suppression of tumorigenicity 2 (sST2), regenerating islet–derived protein 3α (REG3α), tumor necrosis factor receptor 1(TNFR1), interleukin-6 (IL-6), and T cell immunoglobulin and mucin domain–containing protein 3 (TIM3) (11, 14). Of these, sST2 and REG3α have undergone extensive internal or external validation, and algorithms combining these two markers, known as the MAGIC algorithm, have demonstrated predictive value.

Notably, the MAGIC algorithm developed by the Mount Sinai Acute GVHD International Consortium (12) to as Panel 2 in this study, has been most extensively evaluated. It stratifies patients into risk groups either on day 7 post-transplant or at aGVHD onset to predict 6-month NRM. Multiple studies have validated its prognostic utility; furthermore, a randomized multicenter trial adopted the Panel 2 as a stratification criterion for therapeutic intervention (15). However, most of these validations have been conducted in adult Western cohorts, and their applicability to Chinese pediatric populations remains uncertain. Moreover, some external validations for this algorithm have yielded mixed results. One study reported differences in clinical applicability (16). Another found limited prognostic value (17). Additionally, a pediatric cohort analysis indicated that neither sST2 nor REG3α were effective markers for aGVHD diagnosis or prognosis (18).

Building on these advancements, we conducted a prospective multicenter study enrolling Chinese pediatric recipients of allo-HSCT who developed aGVHD. Plasma levels of sST2, REG3α, sTNFR1, IL - 6, and IL - 8 were measured at aGVHD onset. This study aimed to externally validate the MAGIC Consortium’s Panel 2 (sST2 + REG3α) algorithm in a Chinese pediatric multicenter cohort, given its extensive validation in adult and mixed-age populations.

This work represents the first prospective multicenter validation of Panel 2 in this population and explores its potential enhancement through integration of relevant clinical risk factors, with the goal of supporting more individualized post-aGVHD management in pediatric allo-HSCT recipients. In this paper, we describe the biomarker measurements and the determination of the Panel 2 threshold; validate the prognostic utility of Panel 2 by assessing its capacity to stratify patients by 6-month NRM risk and overall survival (OS), as well as to predict treatment response; and evaluate whether incorporating relevant clinical risk factors into the biomarker model could improve prognostic performance. We also discuss the clinical implications, biological rationale, limitations, and directions for future research.

## Methods

### Patient/study population

This multicenter, prospective, observational cohort study consecutively enrolled pediatric patients (aged <18 years) with *de novo* aGVHD following allo-HSCT. The study was conducted at three Chinese tertiary centers: Hunan Children’s Hospital (n=26), Wuhan Children’s Hospital (n=42), and the First Affiliated Hospital of Zhengzhou University (n=36). The enrollment period extended from May 2019 to August 2023. Patients were monitored for aGVHD development for 100 days post-transplantation, with follow-up continuing through March 2024 to assess endpoints: the 6-month cumulative incidence of NRM, OS, and aGVHD treatment response. The study complied with the Declaration of Helsinki and was approved by the Ethics Committee of Hunan Children’s Hospital (Approval No. KY2021 - 55); written informed consent was obtained from all guardians.

### Endpoint and definition

MAGIC criteria were used to diagnose and grade aGVHD (19), and management followed each center’s institutional protocols. Briefly, first-line therapy consisted of glucocorticoids, with second-line agents introduced for suboptimal steroid responses. The primary endpoint was 6-month NRM, NRM defined as death attributable to causes other than underlying disease relapse/progression. Secondary endpoints included treatment response by Day 28 and OS. Complete response (CR) was defined as the complete resolution of aGVHD manifestations in all involved target organs. Partial response (PR) was defined as improvement in symptoms without full resolution in at least one target organ and no worsening in any other. Progressed Disease (PD) was defined as the worsening of acute GVHD in at least one target organ (by at least one stage), with or without concurrent improvement in other organs. Patients were classified as non-responders (NR) if their aGVHD symptoms failed to improve or worsened after systemic corticosteroids, if they required additional systemic immunosuppression for aGVHD, or if they died within the first 4 weeks of treatment. OS was defined as the time from allo-HSCT to death from any cause (censored at last follow-up March 2024).

### Biomarker measurement and panel scoring

Blood samples from each research center were collected in EDTA anticoagulant tubes, transported on ice (4 °C) to a single central laboratory (Guang Zhou BofuRui Biolaboratory) within 48 hours, and centrifuged at 1,500 × g for 10 min at 4 °C to separate serum, which was assayed within 2 hours for sST2, REG3α, sTNFR1, IL - 6, IL - 8 using a customized cytokine detection kit (catalog no. LXSAHM-05) on the Luminex^®^ 200™ system employing xMAP technology according to the manufacturer’s instructions; Predicted 6-month NRM probabilities were obtained using the previously published MAGIC algorithm for Panel 2, as follows: log[−log(1−p)] = −11.263 + 1.844 × log_10_(ST2) + 0.577 × log_10_(REG3α), where *p* denotes the predicted probability of 6-month NRM.

### Statistical analysis

Box plots were used to display the distributions of log-transformed plasma ST2 and REG3α concentrations, as well as their derived Panel 2 scores. Because our assay platforms differed from those used in the original studies, the distribution of Panel 2 scores was also different.

Similar to best practices in predictive modeling (20, 21), we sought to minimize overfitting risk by defining the Panel 2 threshold using the 75th percentile (Q3) of our cohort rather than optimizing cutoffs to maximize performance in the training data. This approach avoids tailoring the model to idiosyncrasies of our sample distribution and ensures broader applicability. Additionally, we examined whether biomarker–outcome associations followed clinically plausible trends, analogous to external parametric trend validation in prior modeling studies, thereby providing further assurance that the selected threshold was not overfit to our dataset.

Continuous variables are presented as median (interquartile range) and compared between the Panel 2 high and low groups by the Mann–Whitney U test. Categorical variables are expressed as number (percentage) and compared by Pearson’s chi-square test or Fisher’s exact test, as appropriate.

We selected commonly recognized clinical prognostic factors and the MAGIC Panel 2 risk group for survival and treatment response analysis. Univariate Cox proportional-hazards models were used to estimate hazard ratios (HRs) and 95% confidence intervals for each variable, and those with P < 0.10 were considered candidates for multivariable modeling. If more than a manageable number of clinical factors met this threshold, a stepwise selection approach was applied to limit the covariate set and avoid overfitting. To preserve model stability and remain consistent with our focus on external validation of the Panel 2 algorithm, no additional exploratory biomarkers were included; only the Panel 2 score together with at most one or two key clinical factors were retained in the multivariable analyses.

Since only three patients experienced relapse, parameter estimates in a competing-risks model would be unstable. Therefore, we treated relapse events as censored and estimated NRM and OS using the Kaplan–Meier method, with differences between groups assessed by the log-rank test and hazard ratios derived from a Cox proportional-hazards model. Day 28 treatment-response rates across the risk groups were visualized with stacked bar charts and compared using the chi-square test or Fisher’s exact test, as appropriate. To evaluate predictive performance, decision-curve analysis (DCA) was conducted for the clinical model, the Panel 2 model, and the combined model to assess net clinical benefit, and receiver operating characteristic (ROC) curves were generated for the same models to compare their discriminative ability.

All analyses were conducted in R version 4.4.1 (R Foundation for Statistical Computing) using the survival and dcurves packages. Two-sided P < 0.05 was considered statistically significant (22).

## Results

### Patient characteristics and transplant overview

This cohort included 105 consecutively enrolled pediatric recipients of allo-HSCT; their baseline characteristics are summarized in **Table 1**. The median age was 7 years (1–18); 78.1% were younger than 12 years and 21.9% were 12 years or older. Underlying diagnoses included non-malignant disorders in 54.3% patients and malignant hematologic diseases in 45.7%. Seventy-six patients (72.4%) received grafts from HLA-mismatched donors, of whom 46 (60.5%) underwent related haploidentical transplantation. Peripheral blood stem cells were the primary graft source in 88.6% of cases; a subset of patients also received supplemental donor bone marrow and/or third-party umbilical cord blood. A large majority received myeloablative conditioning and calcineurin inhibitor-based GVHD prophylaxis.

**Table 1:** Baseline patient characteristics by Panel 2 score dichotomized at the 75th percentile.

Characteristics	Level	All patients (N=105)	Panel 2 H (N=26)	Panel 2 L (N=79)	P Value
Gender	Female	40 (38.1)	9 (34.6)	31(39.2)	0.670
	Male	65 (61.9)	17 (65.4)	48 (60.8)	
Age: yr	<12	82 (78.1)	19 (73.1)	63 (79.7)	0.480
	≥12	23 (21.9)	7(26.9)	16 (20.3)	
Indication for HCT	Malignant disease	48 (45.7)	15 (57.7)	33 (41.8)	0.160
	Non-malignant disease	57 (54.3)	11 (42.3)	46 (58.2)	
RBC compatibility	Matched	56 (53.3)	17 (65.4)	39 (49.4)	0.160
	Mismatched	49 (46.7)	9 (34.6)	40 (50.6)	
HLA compatibility	Matched	29 (27.6)	7 (26.9)	22 (27.8)	0.930
	Mismatched	76 (72.4)	19 (73.1)	57 (72.2)	
Donor type	MRD	7 (6.7)	4 (15.4)	3 (3.8)	0.045
	MUD	25 (23.8)	4 (15.4)	21 (26.6)	
	Haploidentical	50 (47.6)	15 (57.7)	35 (44.3)	
	MMUD	23 (21.9)	3 (11.5)	20 (25.3)	
Graft type	PBSC±BM	31 (29.5)	10 (38.5)	21 (26.6)	0.540
	PBSC+UCB±BM	62 (59.0)	14 (53.8)	48 (60.8)	
	UCB	12 (11.4)	2 (7.7)	10 (12.7)	
MNC count, 10^8/kg	≤10	62 (59.0)	12 (46.2)	50 (63.3)	0.120
	>10	43 (41.0)	14 (53.8)	29 (36.7)	
CD34 count, 10^6/kg	≤10	74 (70.5)	15 (57.7)	59 (74.7)	0.100
	>10	31 (29.5)	11 (42.3)	20 (25.3)	
Conditioning regimen	Myeloablative	83 (79.0)	18 (69.2)	65 (82.3)	0.160
	Reduced	22 (21.0)	8 (30.8)	14 (17.7)	
GVHD prophylaxis	CNI based	82 (78.1%)	18 (69.2.%)	64 (79.0%)	0.210
	PTCy based	23 (21.9%)	8 (30.8%)	15 (21.0%)	
ATG	no-ATG	10 (9.5)	6 (23.1)	4 (5.1)	0.001
	ATG ≤5 mg/kg	43 (41.0)	14 (53.8)	29 (36.7)	
	ATG >5 mg/kg^$^	52 (49.5)	6 (23.1)	46 (58.2)	
Organ distribution	Skin only	43 (41.0)	7 (27.0)	36 (45.6)	0.24
	GI only	42 (40.0)	13 (50.0)	29 (36.7)	
	Liver only	0 (0.0)	0 (0.0)	0 (0.0)	
	≥2 organs involved	20 (19.0)	6 (23.0)	14 (17.7)	
Onset GVHD grade	I–II	89 (84.8)	15 (57.7)	74 (93.7)	**<0.001**
	III–IV	16 (15.2)	11 (42.3)	5 (6.3)	

Acute GVHD manifested at a median of 24 days post-transplant (IQR 15–36 days). Initial severity at diagnosis was grade I: 30 patients (28.6%), grade II: 59 (56.2%), grade III: 15 (14.3%), and grade IV: 1 (0.9%). During follow-up, maximal severity reached grade I: 20 (19%), grade II: 30 (28.6%), grade III: 26 (24.8%), and grade IV: 29 (27.6%) (**Supplementary Table S1**). At 28 days after steroid initiation, 89 (84.8%) achieved complete or partial response (CR/PR), 16 (15.2%) had no response or progressive disease (NR/PD). During subsequent follow-up, 3 patients (2.9%) experienced disease relapse, including 2 (1.9%) relapse-related deaths. At 6 months, 17 patients experienced non-relapse mortality (NRM), corresponding to a cumulative incidence of 16.2%. The aGVHD grade at onset, donor type, and ATG dose in the conditioning regimen differ significantly between the high- and low-Panel 2 groups.

### Association of clinical factor and biomarkers panel with outcomes

**Figure 1:**
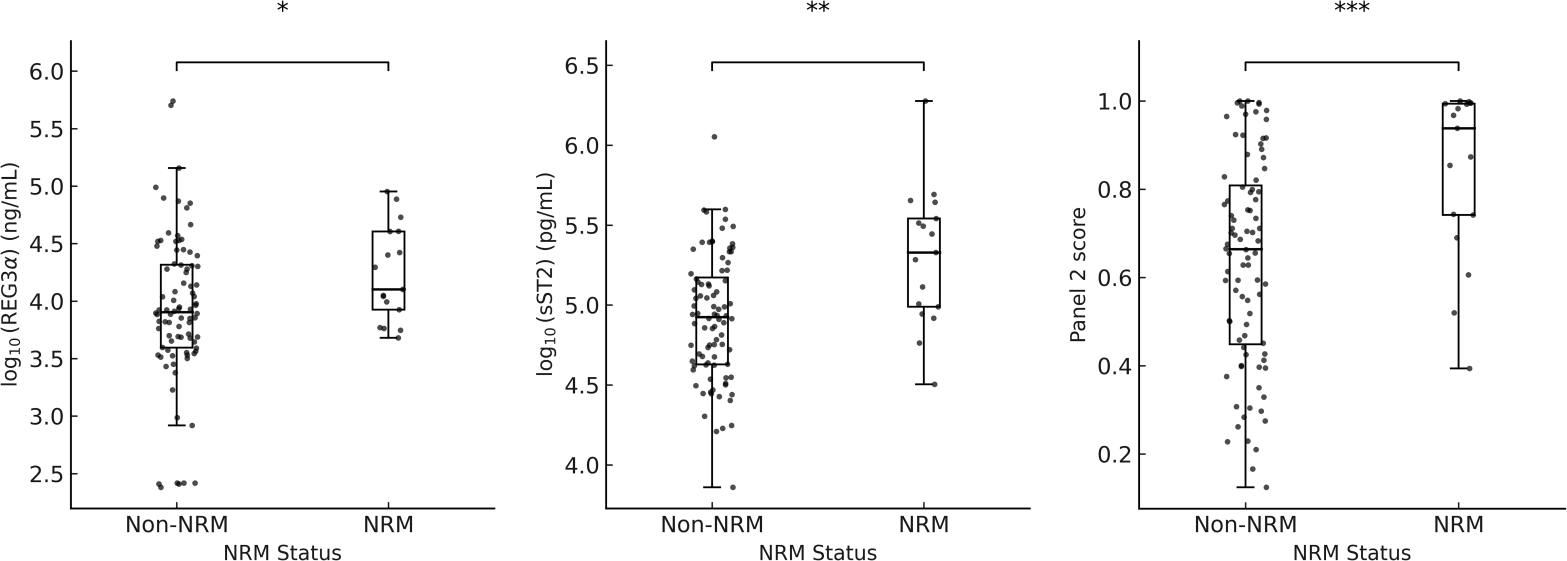
Boxplots of log_10_(REG3a), log_10_(sST2), and panel 2 scores by NRM status. * p = 0.012; ** p = 0.005; *** p = 0.0002.

**Supplementary Figure 1** shows the distributions of log-transformed sST2, REG3α, IL - 6, IL - 8, sTNFR1, and the Panel 2 score. The wide interquartile ranges (IQRs) and presence of extreme values across all biomarkers indicate considerable variability and adequate coverage of clinically relevant value ranges. **Figure 1** shows log-transformed ST2 and REG3α, and panel 2 scores were all significantly higher in the NRM group than in the non-NRM (all P < 0.05). Univariate analysis demonstrated that for clinical variables, patients’ aged ≥12 years and aGVHD grade III–IV at onset were significantly associated with an increased risk of 6-month NRM and poorer overall survival in Cox regression, whereas the stem cell source (PBSC with versus without UBC) was the only factor significantly linked to Day-28 treatment non-response in logistic regression; for Panel 2 score—which were dichotomized at the 75th percentile of Luminex-measured analyte values rather than the fixed clinical MAGIC assay cutoff-the high-risk group had a significantly higher hazard of 6-month NRM (HR 4.26; 95% CI 1.64–10.60; P = 0.003), higher odds of Day-28 non-response (OR 8.0; 95% CI 2.52–25.38; P < 0.001), and worse OS (HR 4.19; 95% CI 2.24–10.34; P = 0.002)(**Table 2**). Exploratory univariate results for the individual biomarkers (sST2, REG3α, TNFα, IL - 6, IL - 8 and sTNFR1) are also provided in **Supplementary Table S2**.

**Table 2:** Univariate analysis of clinical variables and Panel 2 for outcomes.

Variable	Day-180 NRM		Day-28 Response		OS	
	HR (95%)	p-value²	OR	p-value²	OR (95%)	p-value²
Gender						
Male	1					
Female	0.94(0.35 - 2.55)	0.905	1.03(0.34-3.1)	0.956	1.25(0.5-3.11)	0.631
Age						
<12y	1		1			
≥12y	4.29(1.64-11.13)	**0.003**	2.47(0.79-7.57)	0.121	3.43(1.39-8.46)	**0.007**
Indication for HCT						
Malignant disease	1		1		1	
non-malignant disease	1.21(0.46-3.19)	0.695	0.81(0.28-2.36)	0.869	0.94(0.38-2.32)	0.899
RBC Math				0.87		0.87
Matched	1		1		1	
Mismatched	0.62(0.23-1.68)	0.347	0.91(0.31-2.68)	0.869	0.66(0.26-1.68)	0.387
HLA Match						
Matched	1		1		1	
Mismatched	0.94(0.33-2.68)	0.913	1.21(0.36-4.11)	0.76	1.10(0.4-3.06)	0.852
Donor Type				0.95		0.95
MRD	1	0.46	1		1	
MUD	0.29(0.05-1.76)	0.181	0.55(0.04-6.65)	0.635	0.29(0.05-1.76)	0.181
Haploidentical	0.35(0.07-1.68)	0.304	0.90(0.09-9.04)	0.929	0.45(0.1-2.07)	0.304
MMUD	0.59(0.12-3.07)	0.53	0.63(0.05-7.74)	0.719	0.59(0.11-3.05)	0.530
Stem cell Source						
PBSCB±BM+UBC	1		1		1	
PBSC±BM	2.47(0.89-6.81)	0.081	3.68(1.17-11.59)	**0.026**	1.92(0.74-4.97)	0.802
UCB	1.57(0.33-7.56)	0.574	0.82(0.09-7.49)	0.859	1.22(0.26-5.63)	0.802
MNC(10^8/kg)					0.792	
>10	1		1		1	
≤10	1.68(0.59-4.78)	0.328	0.87(0.30-2.54)	0.792	1.20(0.47-3.05)	0.698
CD34(10^6/kg)				0.77		
>10	1		1		1	
≤10	1.43(0.47-4.93)	0.53	1.28 (0.38-4.34)	0.693	0.96(0.36-2.52)	0.928
Conditioning regimen						
myeloablative	1		1		1	
reduced	0.79(0.23-2.76)	0.716	0.48(0.12-2.29)	0.355	0.99(0.33-2.98)	0.983
GVHD Prophylaxis						0.76
CNI Based	1		1		1	
PTCY Based	0.75(0.21-2.6)	0.645	0.45(0.09-2.14)	0.315	0.93(0.31-2.80)	0.897
ATG Dosage						
No used	1		1		1	
≥5mg/kg	1.21(0.27-5.54)	0.802	3.19(0.36-28.18)	0.296	1.45(0.32-6.48)	0.626
< 5mg/kg	0.46(0.09-2.35)	0.347	0.77(0.08-7.68)	0.821	0.45(0.09-2.35)	0.346
GVHD organ distribution						
Skin only	1		1		1	
GI only	1.72(0.56-5.27)	0.347	0.83(0.23-2.98)	0.779	1.94(0.65-5.78)	0.236
≥2 organs involved	1.93(0.52-7.19)	0.327	2.0(0.53-7.57)	0.307	2.41(0.7-8.31)	0.165
GVHD Grad at onset						
I-II	1		1		1	
III-IV	2.75(0.97-7.81 )	**0.058**	2.08(0.58-7.54)	0.263	3.30(1.15-7.98)	**0.025**
Panel 2 Group						
Low	1		1		1	
High	4.26(1.64-10.6 )	**0.003**	8.0(2.52-25.38)	<0.001	4.19(1.7-10.34)	**0.002**

MRD, matched related donor; MUD, matched unrelated donor; MMUD mismatched unrelated donor; CNI based, calcineurin + mycophenolic acid ± methotrexate, PTCY based, Post transplant cyclophosphamide + calcineurin + mycophenolic acid ± methotrexate;ATG, Anti thymocyte globulin; GI, gastrointestinal.

To assess whether age might confound the relationship between aGVHD grade at onset and NRM, we compared age group (<12 vs ≥12 years) with GVHD grade (I–II vs III–IV) using a Pearson χ² test. There was no significant association between age group and GVHD grade (χ² = 0.98; P = 0.32), indicating that age and GVHD grade are independent in our cohort and that age does not act as a confounder of the GVHD grade–NRM relationship.

### Multivariable model for stratification

Variables included in the multivariable Cox model were limited to the pre-specified Panel 2 score and key clinical factors (age and aGVHD onset grade) to help preserve model stability, precluding the addition of other biomarkers. In multivariable analysis for 6-month NRM, OS, and day 28 response.we found that GVHD onset grade was no longer significant for any outcome, whereas age remained an independent predictor of 6-month NRM and OS,but not of day 28 response. The panel 2 score remained significant for every endpoint (**Table 3**). Combining Panel 2 with age yielded four subgroups for further evaluation: Group1: Panel 2 high and age ≥ 12; Group 2: Panel 2 high and age < 12 y; Group 3: Panel 2 low and age ≥ 12 y; Group 4: Panel 2 low and age < 12y.

**Table 3:** Multivarible analysis for outcomes.

Variable	6-Month NRM	p	Day 180 survival	p	Day 28 response	p
	OR (95% CI)		HR (95% CI)		HR (95% CI)	
Age						
<12 y						
≥12 y	4.43 (1.68–11.72)	0.003	3.60 (1.44–9.03)	0.006	2.26 (0.61–8.06)	0.21
Onset GVHD grade						
grade I–II						
grade III–IV	1.80 (0.53–6.08)	0.346	1.96 (0.63–6.12)	0.247	0.74 (0.14–3.37)	0.705
Panel 2 risk group						
Panel 2 Low						
Panel 2 High	3.24 (1.08–9.74)	0.037	3.09 (1.08–8.82)	0.035	8.79 (2.43–34.41)	0.001

Observations: n =105.

R² Nagelkerke: 0.194.

**Figure 2:**
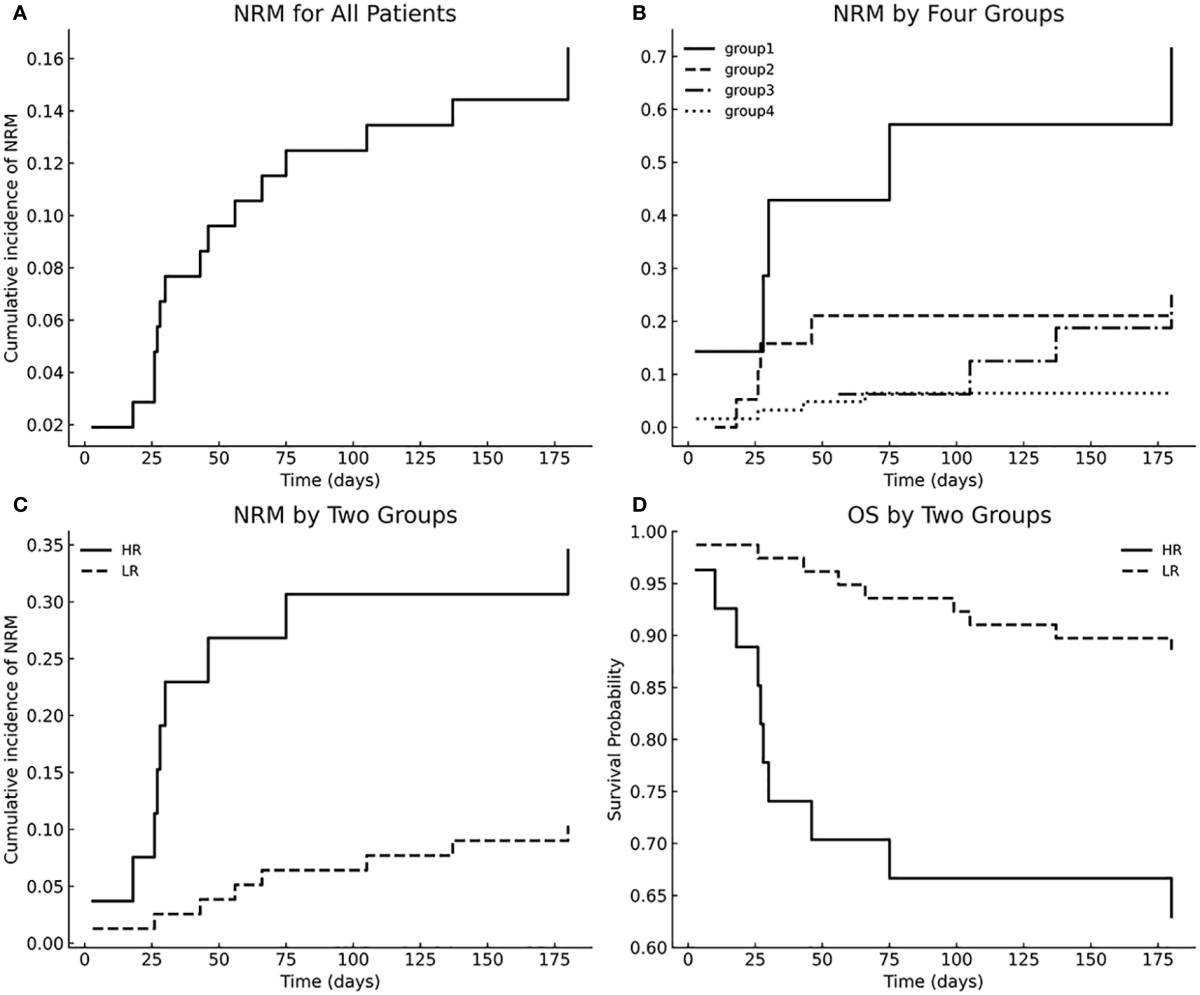
Multivariable model–stratified NRM and OS. **(A)** Cumulative incidence of NRM for all patients; **(B)** NRM stratified by four groups; **(C)** NRM comparison between HR and LR groups; **(D)** Overall survival stratified by HR vs LR groups.

The cumulative incidence of NRM for all patients is shown in **Figure 2A**. To operationalize the models, we first compared 6-month NRM across the four subgroups (**Figure 2B**). Groups 2, 3, and 4 each had significantly lower NRM than Group 1 (all P < 0.01), whereas no significant differences were observed among Groups 2, 3, and 4 (P > 0.05). We therefore combined Groups 2–4 into a single “low-risk” (LR) category, with Group 1 designated “high-risk” (HR). The LR and HR groups had 6-month NRM rates of 12.2% and 71%, respectively; compared with the LR group, the HR group had an HR of 5.0 (95% CI 1.75 - 9.56; P = 0.001) (**Figure 2C**), and their OS rates of 85.7% and 40.0% also differed significantly across strata (P < 0.001) (**Figure 2D**). Therefore, we established a prognostic model based on Panel 2 and patient age.

**Figure 3:**
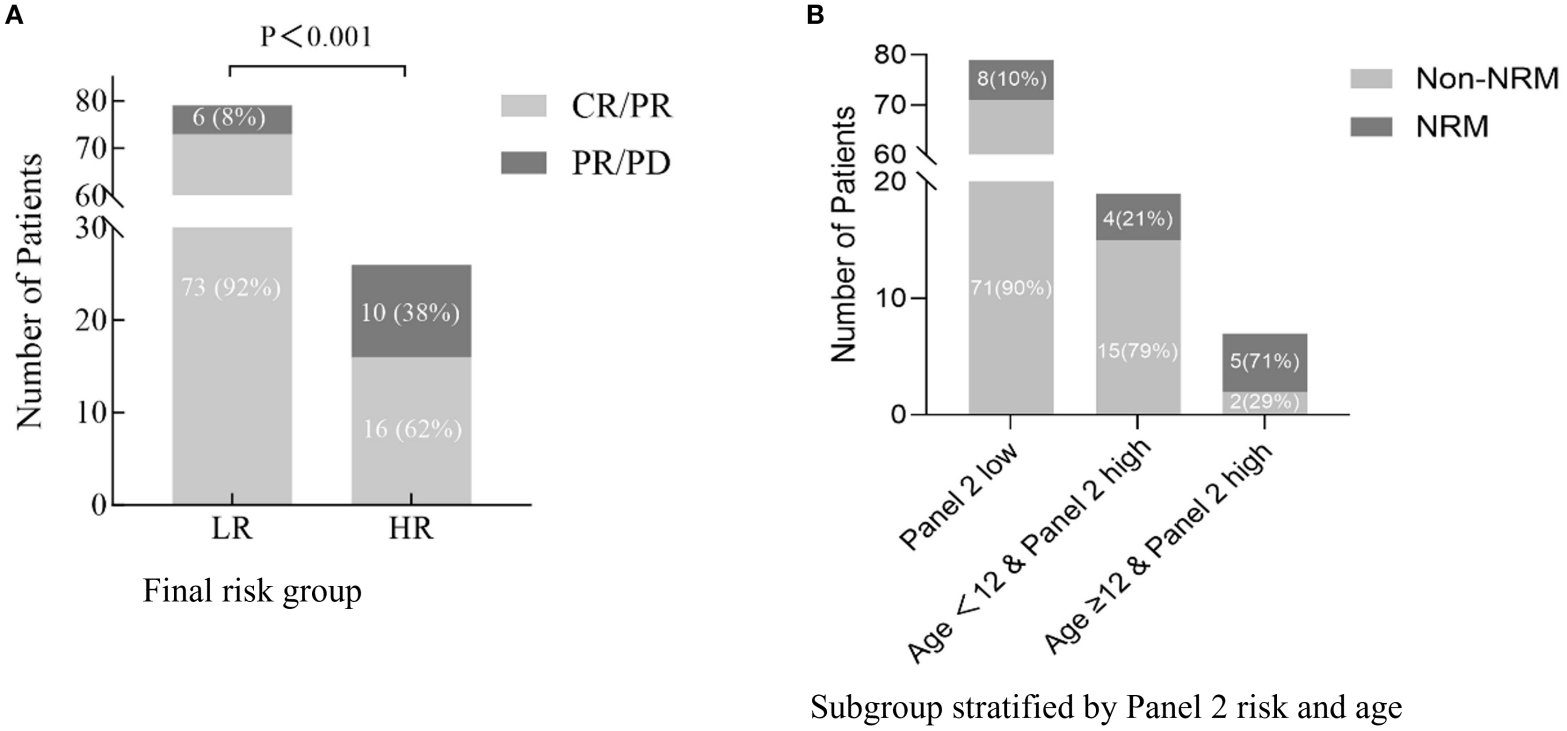
Treatment response and age-modified prognostic impact of Panel 2 in aGVHD **(A)**. Day-28 response to aGVHD therapy (CR/PR vs. PR/PD) stratified by final high-risk (HR) and low-risk (LR) groups. **(B)** Six-month non-relapse mortality (NRM) stratified by Panel 2 risk and age. While patients with high Panel 2 scores generally showed increased NRM, those aged <12 years exhibited substantially lower risk, indicating that age further refines prognostication within the high Panel 2 subgroup.

Since age did not correlate with Day-28 treatment response in univariate analysis, we did not apply our prognostic model to response assessment. Instead, we evaluated Day-28 response using only the Panel 2 classification. The Panel 2 high group had a significantly lower CR/PR rate at Day 28 (62%) compared with the Panel 2 low group (92%) (P < 0.001) (**Figure 3A**).

**Figure 4:**
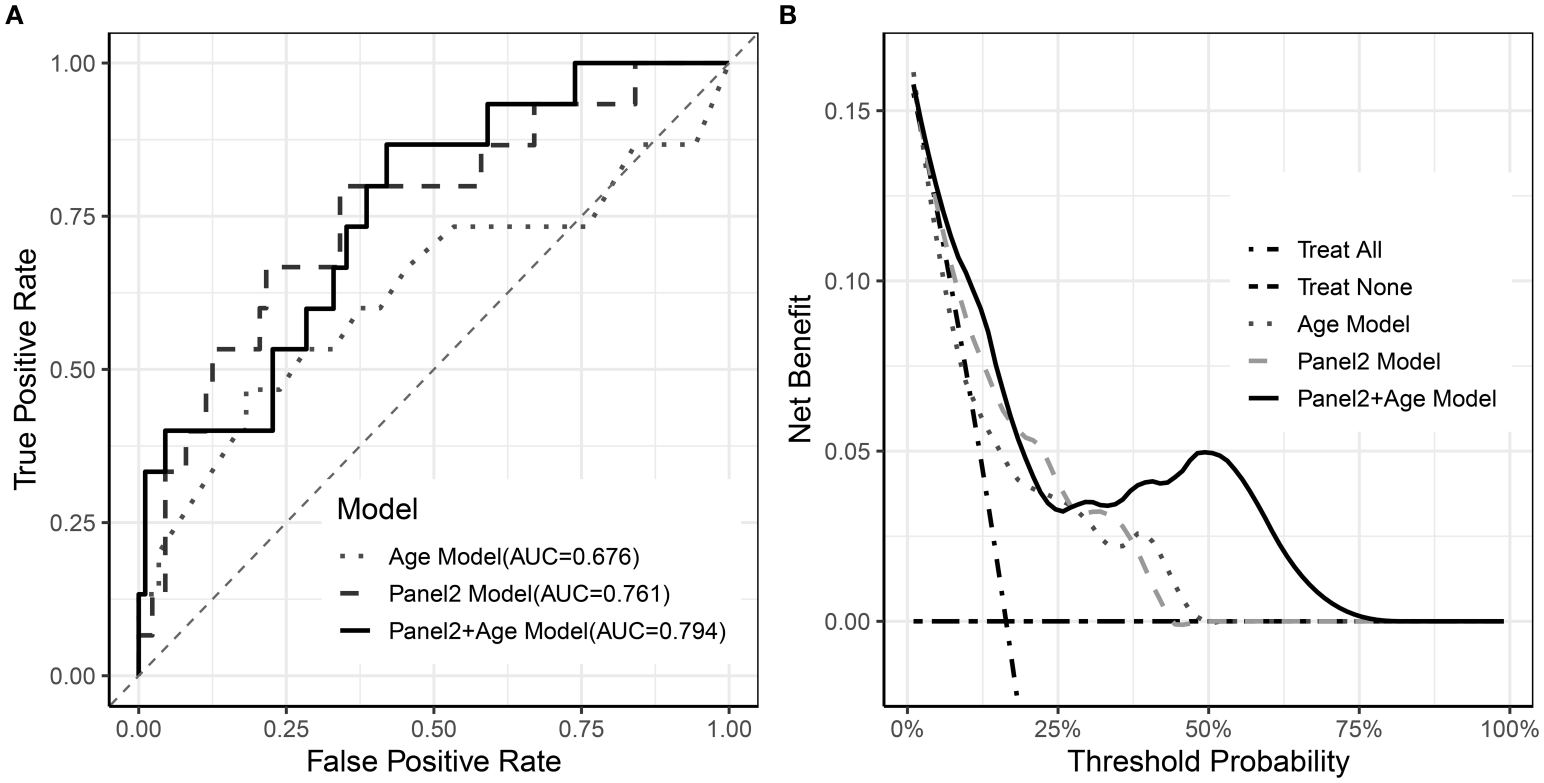
Performance of Age, Panel2, and combined models. **(A)** ROC curves of the three models; **(B)** Decision curve analysis of the three models.

ROC analysis showed that Panel 2 alone yielded an AUC of 0.761 for predicting 6-month NRM, while the addition of age modestly increased the AUC to 0.793 (**Figure 4A**). In contrast, decision-curve analysis revealed a more pronounced difference in clinical utility. Within the clinically relevant threshold range of 10–40%, the combined model (Panel 2 + age) achieved a maximum net benefit of approximately 0.12, compared with 0.05–0.06 for Panel 2 or age alone (**Figure 4B**).

## Discussion

Some studies aim to predict GVHD occurrence and severity at early post-transplant time points, for example, days 7 and 14 post-transplant, while others seek to forecast treatment response and long-term prognosis at GVHD onset or one week after therapy. Early prediction is primarily intended to prevent or mitigate GVHD development; however, once GVHD has occurred, the patient’s condition has already changed, and prognostic assessment after treatment may miss the optimal intervention window. Therefore, this study focuses on the onset time point, as prognostic prediction at onset may have greater clinical translational value. Additionally, several studies have suggested that monitoring dynamic changes in biomarkers is a promising approach (23, 24). However, this method requires more frequent assays and enhanced monitoring, and thus merits further exploration.

In this multicenter, prospective Chinese pediatric allo-HCT cohort, we aimed to validate panel 2, which has been extensively validated and recognized as MAGIC for prognosticating aGVHD in adults, to assess its applicability in our cohort. And we found this panel 2 demonstrated significant prognostic utility for 6-Month NRM, OS and day 28 aGVHD treatment response. These findings align with both adult and pediatric cohorts.

In multivariable analyses of 6-month NRM and OS, both age and the Panel 2 score were independent predictors. However, the aGVHD grade at onset lost statistical significance. The loss of significance for clinical aGVHD grading indicates substantial collinearity with biomarker levels. This finding highlights the superior prognostic utility of Panel 2. Besides, the strong correlation between aGVHD grade and Panel 2 also supports a competitive (or confounding) relationship between traditional clinical assessment and biomarker-based risk stratification in this cohort (**Table 1**).

Among patients with high Panel 2 scores, age further stratified prognosis: those aged ≥12 years had significantly higher 6-month NRM and worse OS. Integrating age into the risk model reclassified 19 of 105 patients (18.1%) originally deemed high-risk as low-risk—potentially sparing them from overtreatment (**Figure 3B**). Conversely, adolescents (≥12 years) with elevated scores represent a subgroup requiring intensified surveillance and earlier therapeutic intervention.

Although ROC curve analysis showed that adding age to Panel 2 yielded only a modest improvement in overall discrimination, decision-curve analysis demonstrated clear incremental clinical utility of the combined model. Within the clinically relevant threshold range, the combined model (Panel 2 + age) achieved a maximum net benefit of approximately 0.12, compared with 0.05–0.06 for Panel 2 or age alone. This corresponds to correctly identifying an additional 6–7 high-risk patients per 100 without increasing unnecessary interventions, underscoring the clinical value of incorporating age into risk stratification beyond Panel 2 alone. In exploratory stratified analyses, the prognostic impact of age appeared most pronounced among patients with high Panel 2 scores, which may explain why the incremental AUC gain was modest when averaged across the full cohort.

In our study, age emerged as an independent predictor of six-month NRM risk with a significance level exceeding initial expectations. To explore potential confounders, we compared baseline characteristics between the two age groups and found no evidence that other variables could account for this difference. This observation stands in contrast to a recent MAGIC Consortium study validating the MAGIC algorithm score as a prognostic biomarker in pediatric GVHD patients, in which age (< 12 vs. ≥ 12 years) did not influence NRM in multivariable analysis (25). Moreover, our review of the Chinese literature revealed no analogous studies of pediatric allo-HSCT cohorts for direct comparison.

The divergence between our findings and those of the MAGIC Consortium may be attributable to differences in baseline clinical characteristics and genetic background. In our cohort, 54% of patients had nonmalignant disease indications, compared to 25% in the MAGIC study. Haploidentical donors were used in 43.8% versus 14%; and ATG at > 5 mg/kg per GIAIC (26)regimen was administered to 52.4% of patients, a rate substantially higher than in the MAGIC cohorts.

To explore why patients aged 12 years or older -those in adolescence within the high-risk Panel 2 subgroup- fared worse, we first note that prior large cohort studies have consistently shown an age effect: adolescents and young adults experience higher rates of acute GVHD compared with younger children, a pattern observed in both unrelated donor (27) and matched sibling donor (28) hematopoietic stem cell transplantation. A plausible biological explanation involves puberty-associated changes in immune regulation. Mature donor T cells are both necessary and sufficient to initiate acute GVHD (29), so the development of GVHD largely depends on antigen presentation and subsequent immune cell activation. Puberty is characterized by marked fluctuations in sex hormones, particularly estrogen, which has been shown to enhance antigen-presenting cell activation via estrogen receptor α–dependent pathways in dendritic cells. This amplifies naïve CD4+ T-cell priming, pro-inflammatory cytokine production, and polarization toward Th1 and Th17 lineages—both implicated in aGVHD pathogenesis (30, 31). These mechanisms not only increase susceptibility to acute GVHD but may also intensify disease severity once GVHD occurs, thereby contributing to the higher NRM observed among adolescents with elevated Panel 2 scores. In contrast, in children under 12 years, the presence of an active thymus supports efficient central tolerance through robust negative selection of donor-derived T cells, even in an inflammatory environment (32), which blunts both the incidence and clinical impact of GVHD despite a high biomarker risk profile.

Our study has several limitations. The limited sample size constrained multivariable modeling and prevented robust evaluation of individual GVHD biomarkers, limiting comparison between single biomarkers and composite algorithms. In addition, to reduce overfitting, we used the third-quartile (Q3) value of the Panel 2 distribution as a pragmatic cutoff, which may not represent the most discriminative threshold. Larger cohorts will be needed to validate biomarker-specific contributions, confirm the prognostic role of age, and refine cutoff selection. Future studies may also explore advanced machine learning approaches, such as the Data Ensemble Refinement Greedy Algorithm (DERGA) (33–35) for biomarker-based risk stratification in aGVHD.

In summary, we validated the prognostic utility of the MAGIC Panel 2 biomarker in a Chinese pediatric cohort. By incorporating age into a multivariable model with Panel 2, we improved the accuracy of predicting 6-month NRM and overall survival following aGVHD. Panel 2 also proved to be a robust predictor of day 28 treatment response. These findings clarify the age-dependent performance of Panel 2, thereby laying the groundwork for tailored risk stratification and therapeutic strategies.

## Data Availability

The raw data supporting the conclusions of this article will be made available by the authors, without undue reservation.
