# Supplementary figures and images for: Age-enhanced MAGIC algorithm predicts mortality in pediatric aGVHD: a multicenter study
Source: Front Immunol. 2025 Sep 12;16:1660861. doi: 10.3389/fimmu.2025.1660861 (PMC12463633; doi:10.3389/fimmu.2025.1660861)

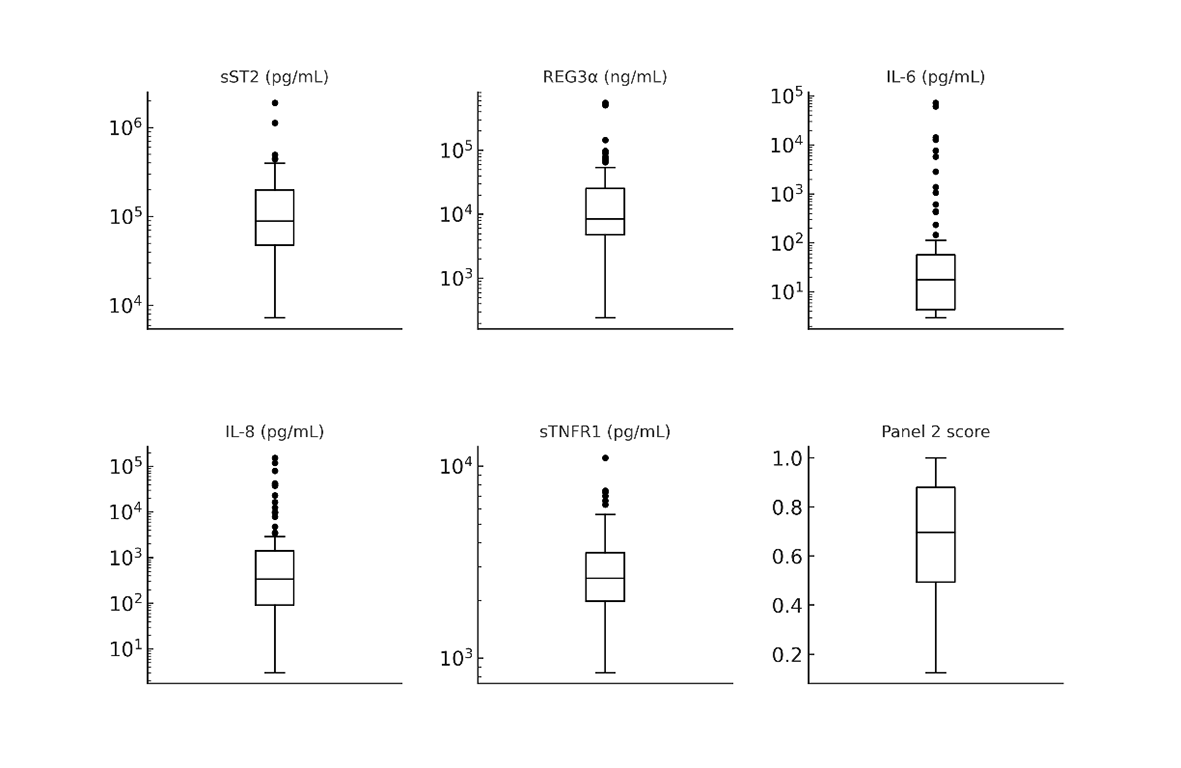

Supplement: Supplementary file 1 [file Image1.tif]
