# Supplementary material for: Age-enhanced MAGIC algorithm predicts mortality in pediatric aGVHD: a multicenter study
Source: Front Immunol. 2025 Sep 12;16:1660861. doi: 10.3389/fimmu.2025.1660861 (PMC12463633; doi:10.3389/fimmu.2025.1660861)
Supplement: Supplementary file 2 [file Table1.docx]

| **Stable S 1. Progression of aGVHD from Initial Grade to Peak Severity** | | | | |
| --- | --- | --- | --- | --- |
| Initial Grade (n=105) | Peak Grade | | | |
|  | 1 (n=20) | 2 (n=30) | 3 (n=26) | 4 (n=29) |
| 1 (n=30) | 17 (56.7%) | 6 (20.0%) | 1 (3.3%) | 6 (20.0%) |
| 2 (n=59) | 3 (5.1%) | 23 (39.0%) | 17 (28.8%) | 16 (27.1%) |
| 3 (n=15) | 0 (0%) | 1 (6.7%) | 8 (53.3%) | 6 (40.0%) |
| 4 (n=1) | 0 (0%) | 0 (0%) | 0 (0%) | 1 (100%) |
|  |  |  |  |  |
