# Supplementary material for: Age-enhanced MAGIC algorithm predicts mortality in pediatric aGVHD: a multicenter study
Source: Front Immunol. 2025 Sep 12;16:1660861. doi: 10.3389/fimmu.2025.1660861 (PMC12463633; doi:10.3389/fimmu.2025.1660861)
Supplement: Supplementary file 3 [file Table2.docx]

| Supplementary Table S2. Univariate analysis of individual biomarkers for outcomes | | | | | | |
| --- | --- | --- | --- | --- | --- | --- |
| Variable | Day-180 NRM | | Day-28 Response |  | OS |  |
|  | HR (95%) | p-value² | OR | p-value² | OR (95%) | p-value² |
| **Log10(sST2)** | 10.19(3.08-33.72) | <0.001 | 15.22(3.02-76.67) | 0.001 | 9.81(3.15 - 30.51) | <0.001 |
| **Log10(REG3α)** | 1.98(0.95-4.12) | 0.067 | 3.81(1.39-3.43) | 0.009 | 1.97 (0.98 - 3.93) | 0.056 |
| **Log10(sTNFR1)** | 22.82(2.8 – 186.09) | 0.003 | 7.31(0.53-101.68) | 0.138 | 16.34 (2.18 - 122.63) | 0.007 |
| **Log10(IL-6)** | 1.03(0.63-1.07) | 0.919 | 0.6(0.28-1.29) | 0.188 | 1 (0.63 - 1.6) | 0.994 |
| **Log10(IL-8)** | 0.78(0.50-1.22) | 0.281 | 0.6(0.35-1.02) | 0.061 | 0.83 0.55 - 1.26) | 0.381 |
